# Human serum-supplemented cell culture conditions improve xenobiotic metabolism in hepatoma cell lines HepG2 and Huh7

**DOI:** 10.1007/s00204-026-04444-6

**Published:** 2026-05-20

**Authors:** Matthias D. Kroesen, Lea Wagmann, Anna-Lena Gehl, Alexandra K. Kiemer, Markus R. Meyer

**Affiliations:** 1 Experimental and Clinical Toxicology and Pharmacology, Center for Molecular Signaling (PZMS), PharmaScienceHub (PSH), Saarland University, Homburg, Germany; 2 Department of Pharmacy, Pharmaceutical Biology, Center for Gender-Specific Biology and Medicine (CGBM), PharmaScienceHub (PSH), Saarland University, Saarbrücken, Germany

**Keywords:** Human serum supplementation, HepG2, Huh7, Xenobiotic metabolism, Cytochrome P450, Synthetic cathinones

## Abstract

Hepatoma cell lines are established in vitro models for xenobiotic metabolism, yet their enzymatic capacity is typically lower than that of primary human hepatocytes. Although widely used as standard culture supplement, fetal bovine serum (FBS) poses ethical concerns and may not provide the optimal nutritional environment for human-specific metabolic functions. This study compared the metabolic activity of the hepatoma cell lines HepG2 and Huh7 cultured in medium supplemented with either human serum (HS) or FBS. Metabolic performance was tested using a dual cytochrome P450 (CYP) substrate cocktail in HepG2, with results compared to literature data for Huh7. For comprehensive evaluation of metabolic capacity, both cell lines were exposed to six synthetic cathinones: 4MeO-αP-VP (4′-methoxy-α-pyrrolidinovalerophenone), 4MeO-αP-BP (4′-methoxy-α-pyrrolidinobutyrophenone), 4MeO-NE-BP (4′-methoxy-*N*-ethylbutyrophenone), 4MeS-αMor-PrP (4′-methylthio-2-morpholinopropiophenone), 4MeS-αP-BP (4′-methylthio-α-pyrrolidinobutyrophenone), and 4MeS-NE-BP (4′-methylthio-*N*-ethylbutyrophenone) under both supplement conditions. HepG2 showed unchanged CYP2D6 activity and increased CYP3A4 activity following HS incubation. HS further led to a general increase in metabolite abundance across both cell lines. Particularly the *N*-oxide formation consistently increased for every compound tested and abundance of most hydroxy and oxo metabolites was higher, most likely due to elevated CYP3A4 activity. Compared to FBS, metabolite formation in the HS group increased significantly, up to fourfold in 12 of 13 reactions for HepG2, and up to fivefold in 8 of 11 reactions for Huh7. Although HepG2 is a more common hepatoma cell line in metabolic research, Huh7 demonstrated higher normalized metabolite formation for most reactions, suggesting it as preferred or highly complementary model. Supplementing culture media with HS significantly enhanced the metabolic competence of the tested hepatoma cell lines. HS can be recommended as a cost-efficient, ethically sound, and physiologically relevant alternative for improving in vitro metabolism assays.

**Supplementary Information:**

The online version contains supplementary material available at 10.1007/s00204-026-04444-6.

## Introduction

The human hepatoma cell lines HepG2 and Huh7 are widely used tools in toxicology research and have retained partial functionality for xenobiotic metabolism (Lin et al. 2012; Sassa et al. 1987). While HepG2 is the typical hepatoma cell line for studying in vitro metabolism (Castell et al. 2006; Fraczek et al. 2013), Huh7 is widely used in studies on hepatitis C virus (HCV) infection and replication, as well as in cancer research (Blight et al. 2002; Dahlem et al. 2022). Both cell lines showed transcription and expression of several phase I and phase II drug-metabolizing enzymes but a cytochrome P450 (CYP) expression and activity lower than 5% than those of primary human hepatocytes (PHHs) (Westerink and Schoonen 2007) (Lin et al. 2012). PHHs remain the gold standard in metabolic research due to maintaining complex, native liver functions (Castell et al. 2006). However, their use is hampered by limited availability and life span, higher preparation effort, and the loss of the functional and morphological phenotype through dedifferentiation (Vinken 2021). Thus, there is the need for hepatoma cells to better simulate human metabolic capacity via optimized nutritional composition as it is a known modulator of metabolic activity. Supplementation of cell media with blood serum or high concentrations of amino acids showed to improve cell differentiation or CYP expression in HepG2 (Boon et al. 2020; Pramfalk et al. 2016). Standard cell culture protocols use fetal bovine serum (FBS) as it is nutrient-rich and supports rapid cell growth. However, its use is raising ethical concerns regarding its production process as byproduct of the meat industry, obtained after slaughtering pregnant cows to get access to the bovine fetus. The fetal blood is then often collected by cardiac puncture without anesthesia from the fetus before further processing (Jochems et al. 2002).

Besides an increased mRNA expression of proteins such as albumin and the hepatocyte nuclear factor 4α, HepG2 also showed increased mRNA expression of CYP3A4 and CYP2D6 after human serum (HS) incubation (Pramfalk et al. 2016). The real-time reverse transcription (RT)-PCR results of Pramfalk et al. were not further investigated on a functional level. Adding 2% HS instead of 10% FBS to Huh7 media resulted in increased mRNA and protein levels of monooxygenases and raised their functional level (Steenbergen et al. 2018). Serum components may influence all molecular levels from DNA to enzyme and potentially activate or act as transcription factors, leading to increased mRNA levels. Furthermore, changes in the amino acid profiles previously showed impact on cell growth and differentiation. Media with a high glycine content induced several signaling pathways, with targets such as the mammalian target of rapamycin (mTOR) or the AMP-activated protein kinase (AMPK) (Boon et al. 2020). Assuming a correlation, more mRNA would lead to increased enzyme levels and an increased metabolite abundance. However, a direct quantitative correlation between transcript levels and functional enzyme activity is not always given (Gry et al. 2009; Payne 2015). Thus, measuring metabolite formation provides a more direct and functionally relevant endpoint for evaluating metabolic capacity.

**Fig. 1:**
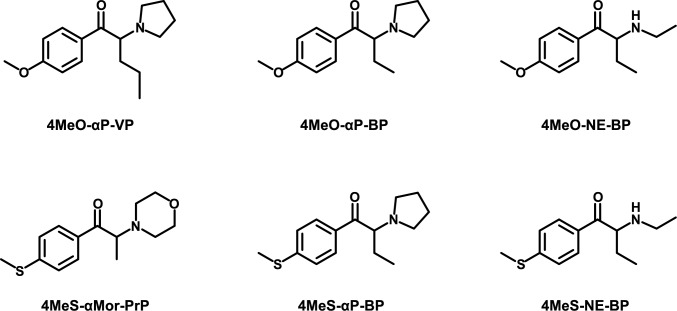
Chemical structures of the synthetic methoxycathinones 4MeO-αP-VP, 4MeO-αP-BP, and 4MeO-NE-BP, and the methylthiocathinones 4MeS-αMor-PrP, 4MeS-αP-BP, and 4MeS-NE-BP

In clinical and forensic toxicology as well as doping control, in vitro assays are essential for identifying potential urinary biomarkers and studying toxicokinetics of drugs and drugs of abuse, including new psychoactive substances (NPS). Suitable in vitro assays should replicate human metabolism while remaining cost-effective. Depending on the supplier and the required effective concentration (e.g. 2% HS versus 10% FBS), HS may represent a more cost-effective alternative to FBS under certain conditions, such as culturing over the same time span. This study aimed to assess the metabolic activity of HepG2 cells via HS supplementation. An improved metabolic activity of HepG2 cells following HS supplementation was anticipated, based on similar findings in Huh7.5 cells (Steenbergen et al. 2018), a subline of Huh7, initially cloned to be highly permissive to replicate subgenomic and genomic HCV RNA (Blight et al. 2002). The formation of metabolites for seven CYP enzymes using a dual-cocktail approach was tested and normalized data were used to allow a direct comparison of the metabolic capacity between HepG2 and Huh7 after supplementation. Given the rapid emergence and rising use of synthetic cathinones (EUDA 2024), we also tested the metabolic activity of each cell line using the six synthetic cathinones (Fig. 1) named following a recommended systematic nomenclature framework (Pulver et al. 2024): 4MeO-αP-VP (4′-methoxy-α-pyrrolidinovalerophenone), 4MeO-αP-BP (4′-methoxy-α-pyrrolidinobutyrophenone), 4MeO-NE-BP (4′-methoxy-*N*-ethylbutyrophenone), 4MeS-αMor-PrP (4′-methylthio-2-morpholinopropiophenone), 4MeS-αP-BP (4′-methylthio-α-pyrrolidinobutyrophenone), and 4MeS-NE-BP (4′-methylthio-*N*-ethylbutyrophenone).

## Materials and methods

### Chemicals and reagents

The six test drugs 4MeO-αP-VP, 4MeO-αP-BP, 4MeO-NE-BP, 4MeS-αMor-PrP, 4MeS-αP-BP, and 4MeS-NE-BP were synthesized as hydrochloride salts following standard procedures (Meltzer et al. 2006). Cathinone stock solutions were prepared in methanol in a concentration of 1 mg/mL and were sterile filtered before cell incubation. HepG2 cells (2 × 10^6^ cells per cryovial) were provided by the German collection of microorganism and cell cultures (DSMZ, Braunschweig, Germany) and Huh7 cells (2 × 10^6^ cells per cryovial) were provided by the Department of Pharmaceutical Biology (Saarland University, Saarbrücken, Germany). Stock solutions of the cocktail substrates dextromethorphan, testosterone, diclofenac, phenacetin, bupropion, amodiaquine, and omeprazole were prepared in a concentration of 100 mM in dimethyl sulfoxide (DMSO). Substrates were separately diluted with FBS and HS supplemented medium, mixed and sterile filtered. Amodiaquine 2HCl, bupropion HCl, diclofenac, omeprazole, phenacetin, phosphate buffered saline (PBS), trypsin, HS from human male blood type AB plasma (sterile-filtered, SKU number H4522, Sigma-Aldrich), Dulbecco’s modified Eagle’s medium (DMEM) high glucose (4.5 g/L, product number D5796), and DMEM low glucose (1 g/L, product number D6046) were obtained from Sigma-Aldrich (Steinheim, Germany), dextromethorphan from Roche (Grenzach, Germany), and testosterone from Fluka (Neu-Ulm, Germany). Water was purified with a Millipore filtration unit (Merck, Darmstadt, Germany), purifying water to a resistance of 18.2 Ω × cm. Trimipramine-d_3_ was from LGC (Wesel, Germany). Penicillin and streptomycin were purchased from Life Invitrogen (Darmstadt, Germany). Acetonitrile, DMSO, methanol, formic acid (LC–MS grade each), ammonium formate (analytical grade), and all other reagents and chemicals (analytical grade) were from VWR (Darmstadt, Germany). 24-well plates and Merck Millex™-GS sterile syringe filter units with mixed cellulose ester (MCE) membranes and 0.22 μm filter pores were purchased from Merck (Darmstadt, Germany). Cell culture flasks (75 cm^2^) were obtained from Sarstedt (Nümbrecht, Germany) and FBS (product number A5256701, LOT 2534396) was purchased from Thermo Fisher Scientific (Dreieich, Germany). Aliquots of the cell lines were cryopreserved and stored in liquid nitrogen at − 160 °C until use.

### Cell culture

Cell culture was performed in a monolayer assay (Figure S1, electronic supplementary material) as described elsewhere (Pramfalk et al. 2016; Steenbergen et al. 2018). After thawing, Huh7 and HepG2 cells were plated on 75 cm^2^ flasks (26,700 cells/cm^2^) in DMEM with high glucose (Huh7) or low glucose (HepG2). DMEM was supplemented with 10% FBS and 1% penicillin/streptomycin (P/S). After reaching an approximate density of 80%, cells of the FBS approach were split every 3–4 days and reseeded at a density of at least 30%. In previous research, media supplementation with 2% HS was determined as most efficient, with higher percentages offering no benefits to cell differentiation (Pramfalk et al. 2016; Steenbergen et al. 2018). Therefore, Huh7 or HepG2 cells of the HS approach were transferred onto a 75 cm^2^ flask in DMEM with 2% HS and 1% P/S after reaching 80% density in FBS supplemented medium. Supplementation with HS results in cell growth arrest after approximately one week. After 3–4 days in HS, cells reached 80–90% confluence. Cells were split once more and Huh7 or HepG2 cells were seeded in 1 mL DMEM with 2% HS and 1% P/S on a 24-well plate with a final concentration of 40,000 cells/cm^2^. After another 4–5 days, cells reached 100% confluence and stopped proliferating in the HS group. HepG2 cells were differentiated in HS for another 14 days and Huh7 for 21 days and cell media was changed every other day. Four days before compound incubation, cells of the FBS approach were transferred onto 24-well plates and incubated until 100% density. Cells were incubated in an incubator (Binder, Tuttlingen, Germany) at 37 °C, 95% air humidity, and 5% CO_2_.

### Incubation of CYP substrate cocktails and synthetic cathinones

Cocktail incubation was performed according to a previous publication with minor modifications (Kroesen et al. 2025b). After cell differentiation, the medium was aspirated and replaced with 250 µL of the compound solution. Cells were incubated separately as technical duplicates and biological quadruplicates with cocktail A (dextromethorphan 9 µM, testosterone 100 µM, phenacetine 12 µM, and diclofenac 4 µM), and cocktail B (bupropion 40 µM, amodiaquine 2 µM, and omeprazole 21 µM) for 24 h. All concentrations were final and the substrate cocktail concentrations were close to their Km values of a validated assay using pooled human liver microsomes (Dinger et al. 2014b). Each cathinone was incubated separately (25 µM) as technical duplicates and biological quadruplicates for 24 h in HS and FBS media, accordingly. Organic solvent in the incubation mixture was below 1% (Chauret et al. 1998). Negative control samples without cells and blank samples without substrates were performed to identify not metabolically formed compounds and to confirm the absence of interfering compounds. After incubation, 50 µL of the cell supernatant were added to 30 µL of acetonitrile (− 20 °C), containing 2.5 µM trimipramine-d_3_ as an internal standard. The mixture was vortexed and centrifuged for 2 min at 18,407 × *g*. A volume of 50 µL of the supernatant was transferred into an autosampler vial and 5 µL were injected onto the liquid chromatography − high-resolution tandem mass spectrometry (LC–HRMS/MS) system.

### Calculation of the relative metabolite formation as an indicator of metabolic activity

The metabolic ratio (MR) was defined as the area ratio of the metabolite to its parent compound measured in full scan (fs) mode (see Eq. 1). Then, the relative metabolite formation was calculated as the ratio of both metabolic ratios in HS and FBS (2). To compare the metabolic activity of HepG2 and Huh7, a normalization factor *f* based on the cell counts was introduced (see Eq. 3). The metabolite formation was calculated as indicated in Eq. (2), multiplied by the normalization factor (see Eq. 4).

1 $${\mathrm{MR}} = { }\frac{{{\text{peak area }}\left( {{\mathrm{metabolite}},{\text{ fs}}} \right)}}{{{\text{peak area }}\left( {{\text{parent compound}},{\text{ fs}}} \right)}}$$

2 $${\text{Metabolite formation}} = \frac{{{\text{MR }}\left( {{\mathrm{HS}}} \right)}}{{{\text{MR }}\left( {{\mathrm{FBS}}} \right)}}$$

3 $${\text{Normalization factor }}f = { }\frac{{10^{5} {\mathrm{cells*mL}}^{ - 1} }}{{{\text{mean cell counts*mL}}^{ - 1} }}$$

4 $${\text{Normalized metabolite formation}} = { }\frac{{{\text{MR }}\left( {{\mathrm{HS}}} \right){*}f}}{{{\text{MR }}\left( {{\mathrm{FBS}}} \right){*}f}}$$

After compound incubation, four random wells of 100% adherent cells in each supplementation group were intensively trypsinized (10 min, 37 °C) and homogeneously resuspended in 1 mL medium. Cell counts were determined with an automated cell counter (Invitrogen Countess 3 FL, Thermo Fisher Scientific, Dreieich, Germany) after injecting 100 µL in a chamber of a cell counting slide. Due to a small sample size, cell counts were compared with a Mann–Whitney U test (n = 4) using the following parameters: unpaired; two-tailed; significance level, 0.05.

### LC-HRMS/MS apparatus and conditions

Apparatus settings and measurement conditions can be found in the electronic supplementary material (ESM, Table S1).

## Results and discussion

In a previous study by Steenbergen et al., CYP specific substrate cocktail incubation after HS supplementation was conducted for Huh7.5 (Steenbergen et al. 2018). In this previous study, Huh7.5 was incubated with CYP1A2, CYP2B6, CYP2C8, CYP2C19, CYP2D6, and CYP3A substrates. Only selected data was shown of the metabolome, with an increased metabolic activity of CYP1A2, CYP2B6, and CYP3A4. In another study, increased mRNA levels of CYP2D6 and CYP3A4 of HepG2 were shown after HS supplementation, but results were not further investigated on a functional level (Pramfalk et al. 2016). Despite the lack of activity of certain CYP enzymes in HepG2, the results of these studies suggested that HS should be considered as a supplement for a range of hepatocyte models to enhance metabolic activity.

### Metabolic activity tested using CYP substrate cocktail incubations in HepG2 cultured in HS or FBS

**Fig. 2:**
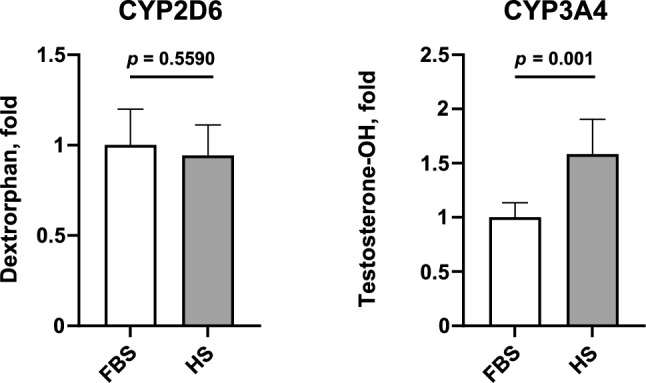
Metabolite formation by CYP2D6 and CYP3A4 in HepG2 cells after incubation for 14 days in 10% fetal bovine serum (FBS) or 2% human serum (HS), respectively; *p*: *t*-test significance level; n = 8 (biological quadruplicates, technical duplicates)

HepG2 were incubated with CYP specific substrates (Dinger et al. 2014a) of CYP1A2 (phenacetin), CYP2B6 (bupropion), CYP2C8 (amodiaquine), CYP2C9 (diclofenac), CYP2C19 (omeprazole), CYP2D6 (dextromethorphan), and CYP3A4 (testosterone). Of the seven substrates, metabolites were only detected of CYP2D6 (dextrorphan, also known as *O*-demethyl dextromethorphan) and CYP3A4 (6β-hydroxytestosterone). In Fig. 2 and according to Eq. (1) in chapter 2.4, the MR of the FBS incubation was calculated and set to 1 as default. The MR of the HS incubation was then compared to the MR of the FBS incubation. Significance of metabolite formation increase within the HS group compared to the FBS group was tested using a *t*-test. The CYP3A4-catalyzed 6β-hydroxylation of testosterone increased more than 1.5-fold when cultured in 2% HS, which was in line with increased mRNA levels shown previously (Pramfalk et al. 2016). As CYP3A4 is responsible for 30–50% of xenobiotic metabolism, the increased metabolic activity would affect a variety of metabolic reactions (Zanger and Schwab 2013). No significant changes in metabolic activity were observed for the CYP2D6-mediated *O*-demethylation of dextromethorphan to dextrorphan, although increased mRNA levels of CYP2D6 after HS supplementation were previously shown (Pramfalk et al. 2016). This underscores the need for studies at a functional level, as correlation coefficients of mRNA and protein levels are often low to moderate (0.2–0.6), probably due to post-transcriptional processes and other complex regulatory mechanisms (Fu et al. 2007; Gry et al. 2009).

No metabolites or interfering compounds were detected in blank samples (no substrate) or negative control (no enzymes). Due to the unknown effects of both sera on protein expression and cell differentiation, cells were quantified by automated cell counting rather than by measuring the total protein amount. By comparing the cell counts of both supplementation groups, differences in metabolite formation due to different cell quantities could be excluded. A Mann–Whitney U test showed no significant difference in the cell counts of both supplementation groups (n = 4). A general increase in metabolic activity of the HepG2 in vitro system was therefore primarily attributed to the increased activity of CYP3A4, as well as to additional monooxygenases not captured by the CYP substrate cocktail. Consistent with published data, no metabolites were formed in HepG2 for most of the investigated CYP enzymes (CYP1A2, CYP2B6, CYP2C8, CYP2C9, and CYP2C19). In other publications using CYP specific substrate cocktails, also only metabolites of CYP2D6 and CYP3A4 were formed, while metabolites of CYP1A2, CYP2B6, CYP2C9, and CYP2C19 could not be detected (Yokoyama et al. 2018). Elsewhere, metabolic activity of HepG2 was only described for CYP3A4 (Stanley and Wolf 2022). However, in another study, metabolites of CYP1A2 and CYP2C8 were detected, additionally to CYP2D6 and CYP3A4 metabolites (Pozo Garcia et al. 2025). These metabolites may not have been detected in this study due to the use of other substrates (CYP2C8) and a different experimental setup.

### Metabolic activity tested using cathinones

In both cell lines, both supplementation groups were exposed to synthetic cathinones for evaluating metabolic activity. Among other NPS, synthetic cathinones are extensively metabolized, which was well-suited to measure the metabolic capacity of the investigated in vitro systems.

### HepG2 with HS or FBS supplementation

**Fig. 3:**
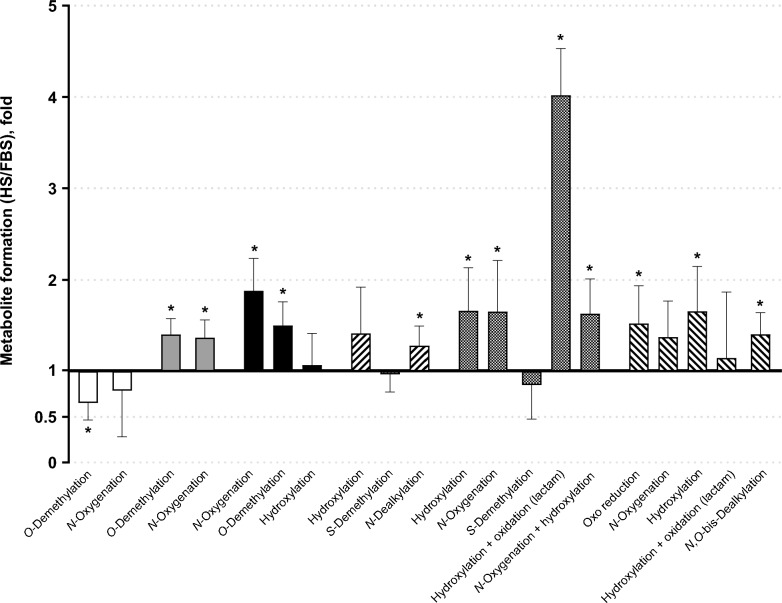
Metabolite formation in HepG2 shown as a ratio of the metabolic ratio of human serum (HS) and the metabolic ratio of fetal bovine serum (FBS). Metabolites of 4MeO-NE-BP (white), 4MeO-αP-BP (grey), 4MeO-αP-VP (black), 4MeS-NE-BP (diagonally upward right pattern), 4MeS-αP-BP (check pattern), and 4MeS-αMor-PrP (diagonally downward right pattern) after incubation for 14 days in 10% FBS or 2% HS. Each bar represents a metabolite of the respective compound. *: *t*-test significance level < 0.05; n = 8 (biological quadruplicates, technical duplicates)

The HepG2 metabolite formation ratio of HS to FBS is shown in Table 1 and Fig. 3, including the mean MR in HS and FBS. Metabolite formation increased significantly in 12 of 13 reactions after HS supplementation. The two-step reaction of hydroxylation + oxidation (oxo formation) of 4MeS-αP-BP increased fourfold and was predominantly catalyzed by CYP3A4, as shown previously (Kroesen et al. 2025a). This observation suggested that the increased CYP3A4 activity after HS supplementation may be functionally potentiated for two-step reactions. As indicated by the results of the CYP substrate incubation, an increased metabolite formation in HepG2 was therefore based on an increased CYP3A4 activity or other monooxygenases not tested in the substrate assay. Metabolites that were previously shown to be formed by CYP2D6 and without CYP3A4 contribution in the monooxygenases activity screening showed no significant change in metabolite formation after HS supplementation, consistent with the results of the CYP substrate incubations. *N*-Oxygenation of 4MeS-NE-BP was only detected in the FBS group and the *O*-demethyl metabolite of 4MeO-NE-BP showed a significantly reduced metabolite formation after HS supplementation. Overall, one additional metabolite was detected in the FBS incubation. This was most likely due to varying monooxygenases expression patterns or an altered substrate affinity after post-translational changes. The abundance of all *N*-oxides was significantly increased after HS supplementation. In a monooxygenases activity screening of the previous study (Kroesen et al. 2025a), CYP3A4 and FMO3 were identified as key enzymes of the *N*-oxygenation of all investigated cathinones. Although not tested in the CYP substrate cocktail, it is expected that FMO3, which is known to catalyze *N*-oxygenations of tertiary amines and the formation of hydroxylamines of primary and secondary amines, was also a key enzyme with elevated activity in this reaction in HepG2. In future studies, metabolic activity should therefore be actively monitored using specific FMO3 substrates. No phase II metabolites were detected in both groups, which could also be due to the already low metabolic ratio of phase I metabolites.

**Table 1:** Metabolic reactions of six synthetic cathinones after exposure to the hepatoma cell line HepG2 cultured in 2% human serum (HS) and 10% fetal bovine serum (FBS). Metabolic reactions are listed in descending order of the metabolite formation ratio of HS to FBS as a fold-change, along with mean metabolic ratios in HS and FBS; n.d.: not detected; N/A: not applicable

Compound	Metabolic reaction	Mean metabolic ratio (MR) in HS	Mean metabolic ratio (MR) in FBS	Metabolite formation ratio, fold (HS/FBS)
4MeO-NE-BP	*N*-Oxygenation	1.31E-04	1.87E-04	0.70
	*O*-Demethylation	5.29E-03	8.81E-03	0.60
4MeO-αP-BP	*O*-Demethylation	1.43E-03	1.02E-03	1.39
	*N*-Oxygenation	5.80E-04	4.30E-04	1.35
4MeO-αP-VP	*N*-Oxygenation	9.10E-04	4.92E-04	1.85
	*O*-Demethylation	8.54E-04	5.75E-04	1.48
	Hydroxylation	3.03E-05	3.06E-05	0.99
4MeS-NE-BP	Hydroxylation	3.00E-03	2.23E-03	1.34
	*N*-Dealkylation	1.62E-03	1.28E-03	1.26
	*S*-Demethylation	2.15E-03	2.02E-03	1.06
	*N*-Oxygenation	n.d	8.39E-05	N/A
4MeS-αP-BP	Hydroxylation + oxidation (lactam)	1.47E-04	3.65E-05	4.02
	Hydroxylation	2.45E-02	1.50E-02	1.64
	*N*-Oxygenation	1.85E-03	1.14E-03	1.63
	*N*-Oxygenation + hydroxylation	1.45E-03	9.12E-04	1.59
	*S*-Demethylation	2.02E-04	2.36E-04	0.85
4MeS-αMor-PrP	Hydroxylation	2.52E-02	1.53E-02	1.65
	Oxo reduction	1.08E-02	7.16E-03	1.51
	*N*-Oxygenation	4.85E-04	3.73E-04	1.30
	*N*,*O*-bis-Dealkylation	5.27E-03	3.85E-03	1.37
	Hydroxylation + oxidation (lactam)	5.92E-05	7.37E-05	0.80

### Huh7 with HS or FBS supplementation

**Fig. 4:**
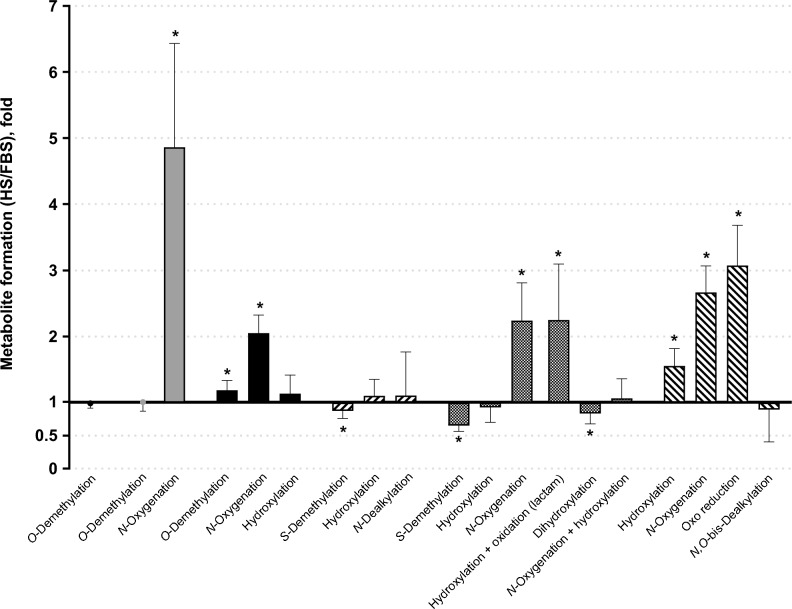
Metabolite formation in Huh7 shown as a ratio of the metabolic ratio of human serum (HS) and the metabolic ratio of fetal bovine serum (FBS). Metabolites of 4MeO-NE-BP (black dot), 4MeO-αP-BP (grey dot and bar), 4MeO-αP-VP (black), 4MeS-NE-BP (diagonally upward right pattern), 4MeS-αP-BP (check pattern), and 4MeS-αMor-PrP (diagonally downward right pattern) after incubation for 21 days in 10% FBS or 2% HS. Each bar represents a metabolite of the respective compound. *: *t*-test significance level < 0.05; n = 8 (biological quadruplicates, technical duplicates)

Metabolite formation increased significantly in 8 of 11 reactions after HS supplementation as shown in Table 2 and Fig. 4. This increased enzyme activity in Huh7 after HS supplementation confirmed data shown for the enhanced activity of CYP-specific metabolic reactions (Steenbergen et al. 2018). In line with the HepG2 incubations, *N*-oxides of all compounds were more abundant after HS supplementation. *N*-Oxygenation of 4MeO-αP-BP increased the most, approximately fivefold. Similar to the fourfold increased oxo formation of 4MeS-αP-BP in HepG2, the 4MeS-αP-BP oxo metabolite approximately doubled in the Huh7 incubation, most likely due to an elevated CYP3A4 activity. The oxo metabolite of 4MeS-NE-BP was only found in the HS incubations. Overall, an additional metabolite was detected in the HS incubation. No phase II metabolites were detected in both groups, which was most likely due to the already low abundance of phase I metabolites.

**Table 2:** Metabolic reactions of six synthetic cathinones after exposure to the hepatoma cell line Huh7 cultured in 2% human serum (HS) and 10% fetal bovine serum (FBS). Metabolic reactions are listed in descending order of the metabolite formation ratio of HS to FBS as a fold-change, along with mean metabolic ratios in HS and FBS; n.d.: not detected; N/A: not applicable

Compound	Metabolic reaction	Mean metabolic ratio (MR) in HS	Mean metabolic ratio (MR) in FBS	Metabolite formation ratio, fold (HS/FBS)
4MeO-NE-BP	*O*-Demethylation	3.75E-03	3.81E-03	0.98
4MeO-αP-BP	*N*-Oxygenation	3.49E-03	6.49E-04	5.38
	*O*-Demethylation	3.03E-03	3.04E-03	1.00
4MeO-αP-VP	*N*-Oxygenation	1.79E-03	8.76E-04	2.04
	O-Demethylation	5.21E-04	4.40E-04	1.18
	Hydroxylation	3.82E-05	3.46E-05	1.11
4MeS-NE-BP	Hydroxylation	7.45E-03	7.00E-03	1.07
	*N*-Dealkylation	8.95E-04	9.56E-04	0.94
	*S*-Demethylation	4.26E-03	4.95E-03	0.86
	Hydroxylation + oxidation (oxo)	3.89E-05	n.d	N/A
4MeS-αP-BP	Hydroxylation + oxidation (lactam)	3.10E-05	1.42E-05	2.19
	*N*-Oxygenation	3.14E-03	1.51E-03	2.09
	*N*-Oxygenation + hydroxylation	7.57E-04	7.31E-04	1.03
	Hydroxylation	2.32E-02	2.62E-02	0.89
	Dihydroxylation	8.53E-05	1.09E-04	0.79
	*S*-Demethylation	2.11E-04	3.45E-04	0.61
4MeS-αMor-PrP	Oxo reduction	2.33E-03	7.66E-04	3.05
	*N*-Oxygenation	1.20E-03	4.49E-04	2.66
	Hydroxylation	4.25E-02	2.76E-02	1.54
	*N*,*O*-bis-dealkylation	2.82E-03	3.68E-03	0.77

### Comparison of the metabolic activity of HepG2 and Huh7 using cathinones

**Fig. 5:**
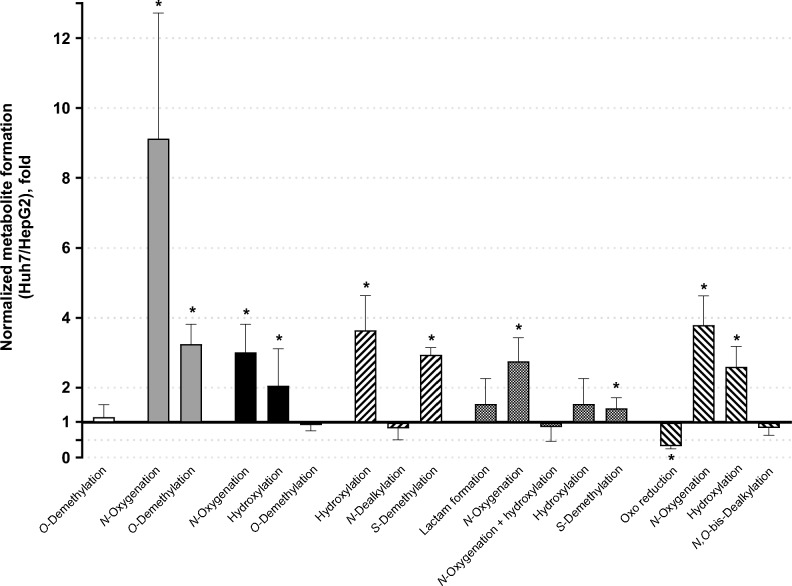
Normalized metabolite formation shown as a ratio of the normalized Huh7 metabolic ratio and the normalized HepG2 metabolic ratio after human serum (HS) supplementation. Metabolites of 4MeO-NE-BP (white), 4MeO-αP-BP (grey), 4MeO-αP-VP (black), 4MeS-NE-BP (diagonally upward right pattern), 4MeS-αP-BP (check pattern), and 4MeS-αMor-PrP (diagonally downward right pattern). Each bar represents a metabolite of the respective compound. *: *t*-test significance level < 0.05; n = 8 (biological quadruplicates, technical duplicates)

**Fig. 6:**
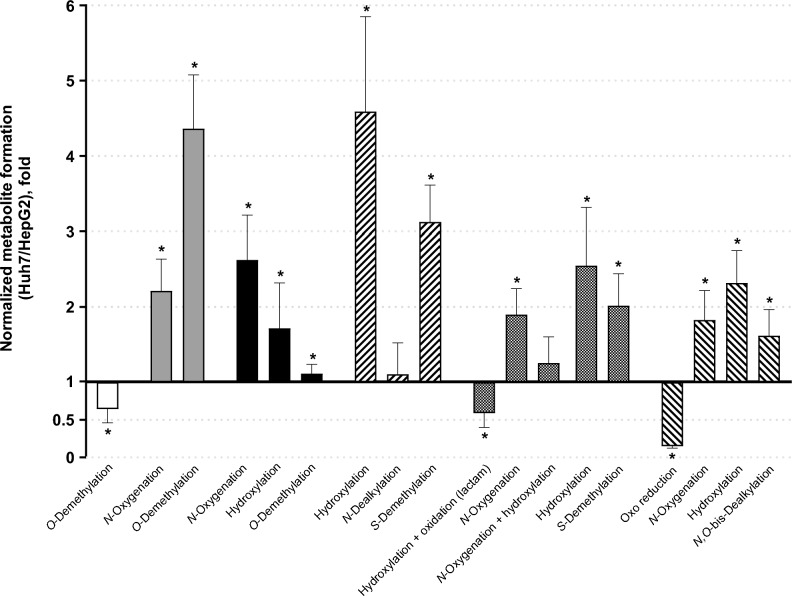
Normalized metabolite formation shown as a ratio of the normalized Huh7 metabolic ratio and the normalized HepG2 metabolic ratio after fetal bovine serum (FBS) supplementation. Metabolites of 4MeO-NE-BP (white), 4MeO-αP-BP (grey), 4MeO-αP-VP (black), 4MeS-NE-BP (diagonally upward right pattern), 4MeS-αP-BP (check pattern), and 4MeS-αMor-PrP (diagonally downward right pattern). Each bar represents a metabolite of the respective compound. *: *t*-test significance level < 0.05; n = 8 (biological quadruplicates, technical duplicates)

Comparing the results for cathinone metabolism of both cell lines, metabolite formation ratios of HS to FBS increased more frequently for HepG2 than Huh7 following HS supplementation (Figs. 3 and 4). This could be due to altered induction across multiple molecular levels. For example, HS may have induced key enzymes or transporters in Huh7 less effectively, leading to a less pronounced increase in the metabolic ratio compared to HepG2. Nevertheless, Huh7 cells frequently exhibited higher metabolic ratios in HS and FBS than HepG2 for their common metabolites (Tables 1 and 2). To compare the metabolic activity of both cell lines, metabolic ratios were normalized. Therefore, cell counts were determined in randomly selected quadruplicates for each cell line under the respective serum condition. The cell counts per mL, mean cell counts, and calculated normalization factor *f* of HepG2 and Huh7 are shown in Table S2 and S3 of the ESM. The resulting normalization factors were 0.27 and 0.40 for HepG2 and Huh7 cultured in HS, respectively, and 0.31 and 0.44 for HepG2 and Huh7 cultured in FBS, respectively. The normalization factors reflected individual growth characteristics, as HepG2 cells tend to grow slightly denser and form multilayered clusters compared to Huh7. Data for normalized metabolite formation are shown in Figs. 5 and 6. Further data on cathinone metabolites, including identified metabolic reactions, mean metabolic ratios in HS and FBS, and metabolite formation ratios (HS/FBS) of the cell lines HepG2 and Huh7 are shown in Tables 1 and 2, respectively. In total, 21 metabolites were identified in the HepG2 incubation and 20 metabolites in Huh7. In comparison to the HepG2 incubations, the hydroxylamine of 4MeO-NE-BP and the lactam of 4MeS-αMor-PrP was not detected in the Huh7 incubations. Instead, the dihydroxy metabolite of 4MeS-αP-BP was only detected in Huh7 in both supplements. Among other reasons, varying monooxygenases expressions and efflux transporter affinities of the metabolites could explain this discrepancy. Of the 18 metabolites formed in both supplements and cell lines, the normalized metabolite formation of Huh7 in HS was significantly higher for 10 of 11 metabolites compared to HepG2 (Fig. 5). In the FBS incubations, the normalized metabolite formation of Huh7 was significantly higher for 13 of the metabolites, decreased for three metabolites and showed no significant change for two metabolites (Fig. 6). Consistent with the results of a higher basal activity of Huh7 compared to HepG2, a higher metabolic activity of Huh7 was shown for CYP1A2 and CYP3A4 in a previous study (Choi et al. 2015). Although HepG2 is far more commonly used in metabolic research, results indicated that Huh7 might be a promising cell line for this purpose. Therefore, Huh7 cells may offer improved in vitro metabolism screening performance compared to HepG2 cells, both in terms of analytes qualitatively detected but also their abundance. However, this advantage is dependent on substrate affinities as well as the expression level of the corresponding monooxygenase in each cell line. The shorter differentiation time of 14 days for HepG2 in HS compared to 21 days for Huh7 is more efficient in terms of time and resources and could be particularly relevant for initial screenings requiring a higher throughput, whereas Huh7 is advantageous for detailed metabolism studies after differentiation. Furthermore, the broader spectrum of monooxygenases activity of Huh7, as shown in previous CYP substrate incubations (Steenbergen et al. 2018) enables more complex in vitro assays on a cellular level, such as CYP inhibition protocols. The characteristics of each in vitro assay are compared in Table 3. Transmembrane transport processes may impact metabolite distributions between the intra- and extracellular compartments and should be considered when comparing cell lines. Importantly, the supernatant reflects the net metabolic output of the cells, combining intracellular metabolic activity with uptake and secretion processes. Therefore, and due to the ease of use, the comparison was limited to the supernatant. Still, for a more comprehensive comparison both compartments should be considered.

**Table 3:** Characteristics of HepG2 and Huh7 in vitro assays, cultured in FBS or HS. → : unchanged; ↑: slight increase, ↑↑: moderate increase, ↑↑↑: strong increase; /: no data. *: Previously discovered (Steenbergen et al. 2018)

	HepG2 (FBS)	HepG2 (HS)	Huh7 (FBS)	Huh7 (HS)
CYP2D6 activity	Low	→	/	/
CYP3A4 activity	Low	↑	Low	↑↑*
*N*-Oxygenation	Low	↑↑	Moderate	↑↑↑
Differentiation time	None	14 days	None	21 days
Costs (effective concentration)	Higher	Lower	Higher	Lower
Ethical justifiability	Complex	Less complex	Complex	Less complex

## Conclusions

This study demonstrated functional evidence that HS supplementation significantly enhanced metabolite formation in both HepG2 and Huh7 cell lines compared to the conventional FBS standard. This indicates that moving to HS, providing a more physiological environment, improves their performance as in vitro metabolism tool. Notably, Huh7 performed better than HepG2, the more frequently used model, in the biotransformation of tested synthetic cathinones. This suggests that Huh7 might not only be a promising alternative but potentially a better tool to study the metabolism of NPS amongst others. Beyond improved metabolic performance and depending on sourcing, processing, supply and effective concentration, the supplementation with HS represents an ethically sound and cost-efficient strategy to FBS. This is in line with the 3Rs principles aiming to reduce reliance on animal-derived components without losing experimental quality. To further characterize these models, future research should use genome, transcriptome, and proteome data to elucidate molecular pathways and signaling cascades that lead to HS-induced metabolic up-regulations. Additionally, more comprehensive studies across diverse chemical classes are needed to further assess the metabolic capacity of Huh7 in comparison with other hepatoma cell lines.

## Supplementary Information

Below is the link to the electronic supplementary material.

Supplementary file1 (PDF 298 kb)

## Data Availability

Data might be made available upon reasonable request from the corresponding author.
