# Supplementary material for: Human serum-supplemented cell culture conditions improve xenobiotic metabolism in hepatoma cell lines HepG2 and Huh7
Source: Arch Toxicol. 2026 May 20;100(9):3977–88. doi: 10.1007/s00204-026-04444-6 (PMC13454003; doi:10.1007/s00204-026-04444-6)
Supplement: Supplementary file 1 [file 204_2026_4444_MOESM1_ESM.pdf]

Electronic Supplementary Material

Human Serum–Supplemented Cell Culture Conditions Improve Xenobiotic Metabolism in Hepatoma Cell Lines HepG2 and Huh7

*Matthias D. Kroesen[1], Anna-Lena Gehl[1], Lea Wagmann[1], Alexandra K. Kiemer[2], Markus R. Meyer[1]**

[1]Experimental and Clinical Toxicology and Pharmacology, Center for Molecular Signaling (PZMS), PharmaScienceHub (PSH), Saarland University, Homburg, Germany

[2]Department of Pharmacy, Pharmaceutical Biology, Saarland University, Saarbrücken, Germany; PharmaScienceHub (PSH); Center for Gender-Specific Biology and Medicine (CGBM)

**Correspondence:* markus.meyer@uni-saarland.de

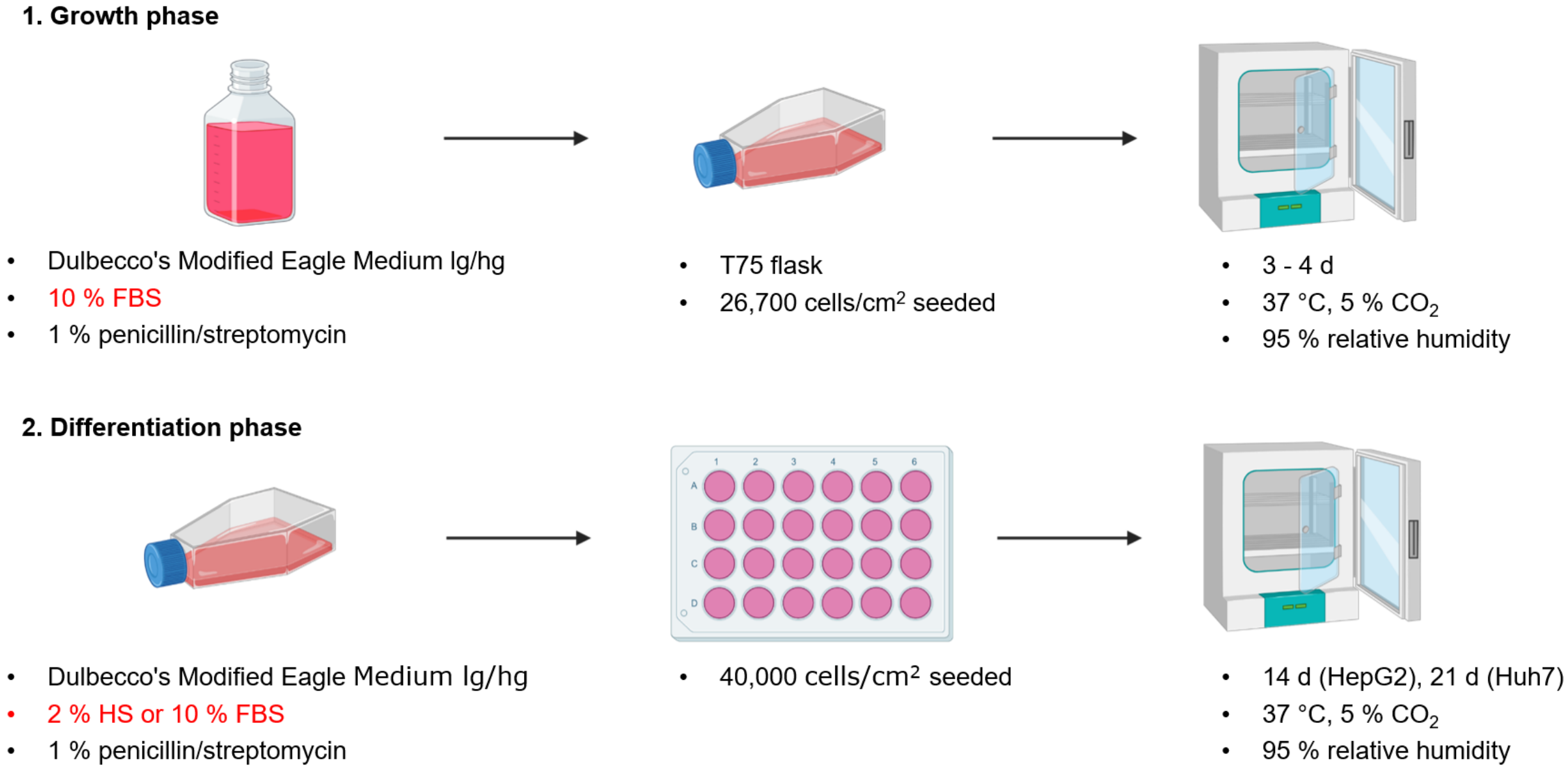

Figure S1. Cell culture protocol of HepG2 and Huh7. Cells were grown for 3–4 days in 10 % fetal bovine serum (FBS), followed by a differentiation phase of 14 days (HepG2) or 21 days (Huh7) in 2 % human serum (HS); lg: low glucose (HepG2), hg: high glucose (Huh7).

LC-HRMS/MS apparatus and conditions

A Thermo Fisher (TF, Dreieich, Germany) Dionex UltiMate 3000 RS pump consisting of a degasser, a quaternary pump, and an UltiMate 3000 RS autosampler, coupled to a TF Q Exactive Plus system equipped with a heated electrospray ionization (HESI)-II source were used. A mass calibration was done according to the manufacturer's recommendations using external mass calibration prior to analysis. Gradient elution was performed on a TF Accucore PhenylHexyl column (100 mm x 2.1 mm, 2.6 µm) with a 2 mM aqueous ammonium formate solution containing 0.1 % (*v/v*) formic acid (pH 3, eluent A) and 2 mM ammonium formate solution in acetonitrile/methanol (50:50, *v/v*) containing 0.1 % (*v/v*) formic acid, and 1 % (*v/v*) water (eluent B). The gradient was stepped as follows: 0–2.5 min hold 99 % A, 2.5–8 min to 1 % A, 8–9.5 min hold 1 % A, and 9.5–11.5 min hold 99 % A. Initial flow rate from 0–9.5 min was 500 µL/min and final flow rate was 800 µL/min from 9.5–11 min. Injection volume was 5 µL for every sample. Mass spectrometry was performed using full scan data and a subsequent data-dependent acquisition (DDA) with priority to mass-to-charge ratios (*m/z*) of parent compounds and their expected metabolites. The inclusion list contained *m/z* values of likely formed metabolites such as *O*- or *S*-demethyl, hydroxy, and oxo reduction metabolites (phase I) as well as sulfates, glucuronides (phase II), and combinations thereof. Chemdraw 23.1.1 was used to draw structures of expected metabolites and for exact mass calculations. TF Xcalibur Qual Browser software version 4.6 (Dreieich, Germany) was used for data handling, with the following settings for peak integration: peak detection algorithm: INCOS; baseline window: 40; area noise factor: 5; peak noise factor: 10. Mass deviations of the parent compound were accepted up to 5 ppm. All samples were measured in positive and negative ionization mode. Instrument settings, data generation settings, and data evaluation settings are shown in **Table S1**.

Table S1. Instrument settings for analysis using the TF Q Exactive Plus system.

**HESI-II source conditions**	
Heater temperature	320 °C
Ion transfer capillary temperature	320 °C
Spray voltage	4.0 kV
Ionization mode	Positive or negative
Sheath gas	60 arbitrary units (AU)
Auxiliary gas	10 AU
Sweep gas	0 AU
S-lens RF level	50.0
**Full scan data acquisition**	
Resolution	35,000
Microscans	1
Automatic gain control (AGC) target	1e6
Maximum injection time (IT)	120 ms
Scan range	*m/z* 50-750
**Settings for DDA mode plus inclusion list**	
Option "pick others"	Enabled
Dynamic exclusion	0.1 s
Resolution	17,500
Microscans	1
Isolation window	1.0 *m/z*
Loop count	5
AGC target	2e5
Maximum IT	250 ms
High collision dissociation cell with stepped normalized collision energy	17.5, 35.0, 52.5
Exclude isotopes	On
Spectrum data type	Profile
Underfill ratio	1 %

Table S2. HepG2 and Huh7 cell counts/mL in the human serum incubations after trypsinization (n=4), mean cell count and calculated normalization factor *f* of HepG2 and Huh7.

	HepG2 cell count/mL	Huh7 cell count/mL
	3E5	4E5
	4E5	3E5
	6E5	1E5
	2E5	2E5
Mean cell count	3.75E5	2.5E5
Normalization factor *f*	0.27	0.40

Table S3. HepG2 and Huh7 cell counts/mL in the fetal bovine serum incubations after trypsinization (n=4), mean cell count and calculated normalization factor *f* of HepG2 and Huh7.

	HepG2 cell count/mL	Huh7 cell count/mL
	5E5	2E5
	3E5	3E5
	3E5	2E5
	2E5	2E5
Mean cell count	3.25E5	2.25E5
Normalization factor *f*	0.31	0.44
